# Somatostatin Receptor-Based Molecular Imaging and Therapy for Neuroendocrine Tumors

**DOI:** 10.1155/2013/102819

**Published:** 2013-09-11

**Authors:** Ling Wang, Kun Tang, Qi Zhang, Huanbin Li, Zhengwei Wen, Hongzheng Zhang, Hong Zhang

**Affiliations:** ^1^Department of Nuclear Medicine, The Second Affiliated Hospital of Zhejiang University School of Medicine, Hangzhou, Zhejiang 310009, China; ^2^Department of Nuclear Medicine, Wenzhou Medical University, Wenzhou, Zhejiang 325003, China; ^3^Zhejiang University Medical PET Center, Zhejiang University, Hangzhou, Zhejiang 310009, China; ^4^Institute of Nuclear Medicine and Molecular Imaging, Zhejiang University, Hangzhou, Zhejiang 310009, China; ^5^Key Laboratory of Medical Molecular Imaging of Zhejiang Province, Hangzhou, Zhejiang 310009, China; ^6^Department of Radiology, The First Affiliated Hospital of Wenzhou Medical University, Wenzhou, Zhejiang 325000, China

## Abstract

Neuroendocrine tumors (NETs) are tumors originated from neuroendocrine cells in the body. The localization and the detection of the extent of NETs are important for diagnosis and treatment, which should be individualized according to the tumor type, burden, and symptoms. Molecular imaging of NETs with high sensitivity and specificity is achieved by nuclear medicine method using single photon-emitting and positron-emitting radiopharmaceuticals. Somatostatin receptor imaging (SRI) using SPECT or PET as a whole-body imaging technique has become a crucial part of the management of NETs. The radiotherapy with somatostatin analogues labeled with therapeutic beta emitters, such as lutetium-177 or yttrium-90, has been proved to be an option of therapy for patients with unresectable and metastasized NETs. Molecular imaging can deliver an important message to improve the outcome for patients with NETs by earlier diagnosis, better choice of the therapeutic method, and evaluation of the therapeutic response.

## 1. Introduction

Neuroendocrine tumors (NETs) are unique tumors that originate almost everywhere in the body from neuroendocrine cells [1]. They have secretory granules which can produce biogenic amines and polypeptide hormones [2]. All these tumors share the features of the neuroendocrine cell system [3]. NETs have particular characteristics including low incidence, low proliferation rate, and sometimes the hyper secretion of biologically active substances [4]. The diagnosis of lesion is limited because it has slow metabolic rate, small size, and various localization [5]. Those NETs of unknown primary may have a relatively favorable prognosis [6]. The primary sites in gastrointestinal and bronchopulmonary tracts are most frequent [7]. Gastroenteropancreatic neuroendocrine (GEP-NET) tumors are neoplasms with variable clinical expressions. They produce and secret various amines and peptides, which can be used as tissue and circulating markers [8], representing approximately 2% of all gastrointestinal tumors [9]. Pheochromocytomas are malignant in approximately 10% of patients. The histology of benign and malignant tumors has no obvious differentiation. The malignant tumors are diagnosed by the presence of metastatic lesions or recurrence [10].

Overall, 5- and 10-year survival rates of NETs were 78 and 63%, respectively [11]. There are various clinical behaviors of NETs. They may have a function or not. The clinical use of specific radiolabeled ligands for imaging and therapy is based on the presence of peptide receptors and transporters at the cell membrane and the neuroamine uptake mechanisms of NETs. Because the majority of NETs express somatostatin receptors (SSTR) which bind to somatostatin (SST), they can be successfully targeted [2]. The understanding and diagnosis of NETs have been greatly improved by morphologic and functional imaging modalities [12]. This paper is a systematic review about the somatostatin receptor-based molecular imaging and therapy for NETs.

## 2. SST and SSTR

SST is produced by neuroendocrine, immune, and inflammatory cells in response to many kinds of factors, such as ions, nutrients, neuropeptides, neurotransmitters, and thyroid [13]. It is present in the cerebral cortex, the brain stem, the hypothalamus, the pancreas, and the gastrointestinal tract. It is a cyclic and regulatory peptide consisting of 14 amino acids [13, 14]. A family of G-protein-coupled receptors mediates the function of SST which comprises five distinct subtypes (characterized SSTR1-5) [13, 15]. The SSTR subtypes overexpressed in NETs are related to the type, origin, and grade of differentiation of tumor [16]. A number of different tumors have receptors for SST [17]. SSTR expresses in various regions such as the brain, the adrenals, the pancreas, and the gastrointestinal tract [18]. SSTR also distributes in tumor tissues of neuroendocrine origin. SSTR is overexpressed in various human cancers. The overexpression of SSTR is a characteristic of NETs, which can be used to localize the primary tumor and its metastases by imaging with the radiolabeled SST analogues [19]. Receptor targeting with subtype-specific radiolabeled SST analogues is based on the structural differences between SSTR subtypes. The imaging of the SST subtype 2 (SST_2_) overexpressing NETs has been developed and has had extensive clinical applications for almost two decades [20].

There is significant variation in SSTR subtype expression between the tumors of the same type [21]. The majority of tumors expressed SSTR types 1, 2, 3, and 5, and a minority expressed SSTR4 [22]. The expression of SSTR2 on pancreatic endocrine or carcinoid tumors is predominant [21]. The clinical use of SST is limited because it has a short half-life (about 2 minutes) in plasma [23]. SST analogues used to evaluate the use and effectiveness of the management in NETs patients have been synthesized widely. The radioisotopes used in nuclear medicine both for imaging and therapy are showed in Table 1. Imaging with SST analogues is considered as imaging method of first choice for NETs because it has high specificity, low antigenicity, rapid clearance, and good tissue penetration [24]. Octreotide and octreotate are widely used as SST analogues, and the role of SST analogues in the clinical use is well established. This review summarizes the clinical use of SSTR imaging and therapy in NETs as well as the use of SSTR as a platform for gene report imaging.

## 3. The Clinical Use of Radioisotope Labeled SSTR in NETs

### 3.1. Somatostatin Receptor Imaging (SRI)

SRI is widely used for the diagnosis, as well as staging and restaging of NETs [25]. NETs are usually diagnosed by a combination of clinical symptoms, histology, and hormonal excess. After diagnosis of NET is established, a search for its localization is carried out using common morphologic imaging methods in the past [26]. However, it is difficult to use conventional imaging techniques to map the lesions accurately. There is an urgent need to establish better imaging modalities to detect the lesions for NETs. The tracers used for SPECT and PET in NETs are showed in Table 2.

### 3.2. Single Photon Emission Computed Tomography (SPECT) Imaging

^111^In-pentetreotide used to be the first choice for the visualization of receptor for SST analogues. Tumors and metastases that express the SSTR subtypes SSTR2, SSTR3, or SSTR5 can also be visualized in vivo after injection of ^111^In-pentetreotide [27]. It differentiates scar tissue from tumor recurrence after the pituitary surgery or radiotherapy. Another agent, [^111^In-DTPA(0)]octreotide (^111^In-DTPAOC), is also a tracer of a great potential use for the imaging of SSTR-positive tumors. ^111^In-DTPAOC scintigraphy is also a scintigraphy modality of choice for NETs. Still, there are patients in whom imaging findings are negative or weak positive [28]. ^111^In-DOTATOC is reported to be of great value for the diagnosis of patients with octreotide receptor-positive tumors [29]. The efficacy of scanning with ^123^I-octreotide was evaluated by localizing tumors in 42 patients with NETs. It was found that those often unrecognized primary tumors or metastases were visualized in 12 of 13 patients with carcinoid tumors as well as in 7 of 9 patients with endocrine pancreatic tumors.

There is an overall high sensitivity of SRI to localize NETs. The value of SRI in patients with NETs has been proven [17, 30, 31]. The scintigraphy provides important information in NET patients and has a strong impact on further therapeutic management [31]. Both positive and negative results of SRI are very useful; the former may predict the effect of octreotide therapy to NETs [17].

### 3.3. Positron Emission Tomography (PET) Imaging

**Figure 1:**
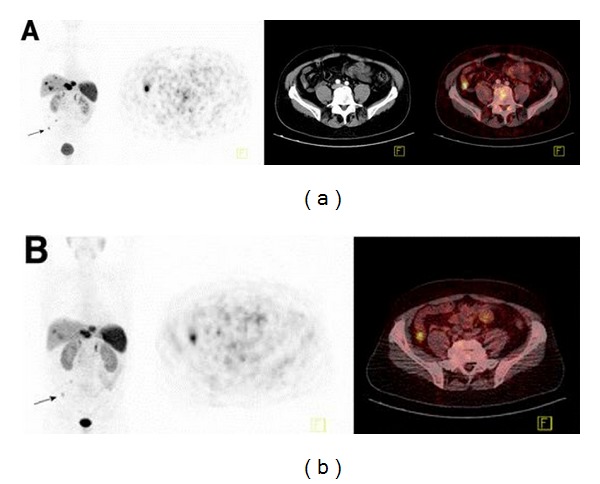
Lesions have exclusive higher uptake in ^68^Ga-DOTATOC than ^68^Ga-DOTATATE imaging. (a) From left to right: ^68^Ga-DOTATOC PET maximum-intensity projection, ^68^Ga-DOTATOC PET, CT, and PET/CT fusion. (b) From left to right: ^68^Ga-DOTATATE PET maximum-intensity projection, ^68^Ga-DOTATATE PET, and PET/CT fusion. The arrow refers to ileal carcinoid (SUVmax ^68^Ga-DOTATOC, 21.0; SUVmax ^68^Ga-DOTATATE, 8.2) [32].

^68^Ga-labeled somatostatin analogues are widely used [2]. The two compounds frequently used in functional PET imaging are ^68^Ga-DOTA (0), Tyr (3) octreotate (^68^Ga-DOTATATE) and ^68^Ga-DOTATOC [32]. ^68^Ga-DOTATATE PET/CT is a useful imaging modality for NETs. In 38 patients, the sensitivity is 82% [33]. Another study of 18 patients with pulmonary NETs showed that all typical carcinoids showed a high uptake of ^68^Ga-DOTATATE [34]. The comparison of the ^111^In-DTPAOC SPECT and ^68^Ga-DOTATOC PET shows that the latter is superior in detecting small lesions with low tracer uptake [35]. In another comparison study of 84 patients with known or suspected NETs, ^68^Ga-DOTATOC PET shows a significantly higher detection rate compared with SPECT and diagnostic CT [36]. A study of 40 patients with metastatic NETs who underwent ^68^Ga-DOTATOC and ^68^Ga-DOTATATE PET/CT reported that the two had almost the same accuracy for the diagnosis of NET lesions; however, standard uptake value (SUV) max of ^68^Ga-DOTATOC scans is higher than ^68^Ga-DOTATATE (Figure 1) [32]. It was also reported that the diagnostic value of PET/CT with ^68^Ga-DOTATATE and ^68^Ga-DOTATOC in the same patients with GEP-NET is almost the same, but the maximal uptake of ^68^Ga-DOTATATE tended to be higher than ^68^Ga-DOTATATE [37].

A case reported that, in a patient who had synchronous colorectal cancer and pancreatic NET, the ^68^Ga-DOTATATE PET and ^18^F-FDG PET imaging showed two different tumor types within the liver metastases. This case suggested that combinational ^68^Ga-DOTATATE PET and ^18^F-FDG PET imaging modalities are of potential use in understanding the biology of the NETs and managing the NETs [38].

Several novel agents have been developed. DOTANOC is the first compound for PET imaging and is reported to have a higher affinity for SSTR2 as well as for SSTR5 [39]. The first in-humans study with ^64^Cu-DOTATATE imaging had an excellent imaging quality, reduced radiation burden, and increased lesion detection rate when compared with ^111^In-DTPA-octreotide. It identified additional lesions in 6 of 14 patients (43%) [40]. ^18^F-fluoropropionyl-Lys0-Tyr3-octreotate (^18^F-FP-Gluc-TOCA), another new carbohydrate analog of octreotide, is under research [41]. In 25 patients with different SSTR-positive tumors, ^18^F-FP-Gluc-TOCA showed a fast and intense tumor accumulation and a rapid clearance from blood serum [42].

A study concluded the sensitivity and specificity of SSTR PET or PET/CT in detecting thoracic and/or GEP-NETs, which were 93% and 91%, respectively [43]. PET resulted in a modified restage in 12 patients (28.6%), while the treatment plans were affected in 32 patients (76.2%). It prevented unnecessary surgery in six patients, while two patients with lesions that did not express SSTR were excluded from PRRT [44]. ^68^Ga-DOTANOC PET/CT can affect the tumor staging and modify the treatment in more than half the patients [19]. To predict the therapy response earlier in tumors is essential to guide the therapy and at the same time avoid the side effects and lower the costs caused by ineffective therapies [45]. However, using conventional imaging techniques and response criteria to assess treatment response is often complicated [12]. Decreased ^68^Ga-DOTATATE uptake in lesions after the first cycle of PRRT correlated with clinical symptoms improvement and predicted time to progression in well-differentiated NET patients [45].

### 3.4. Somatostatin Receptor Targeted Radionuclide Therapy (SRTRT)

The treatment of the NETs includes peptide receptor therapy, somatostatin analogues, and surgery [28]. Surgery is still the therapy of first choice, while the vast majority of NETs will need further treatment with SST analogues and/or interferon [46]. There are few treatment options for those metastasized or inoperable endocrine GEP tumors. Chemotherapy for those NETs may be effective, but the response usually lasts less than one year [47]. The predominant expression of SST_2_ receptors in NETs is essential for the application of radiolabeled octapeptide SST analogues [21], as well as for PRRT using ^90^Y- and ^177^Lu-DOTATATE/DOTATOC [48]. The radiological response was measured with response evaluation criteria in solid tumors (RECIST) criteria [49]. SSTR PET imaging, including the common tracer ^68^Ga-DOTATOC, is becoming the basis of the selection of candidates for PRRT [50]. Patients with high ^68^Ga-DOTATOC uptake (SUV > 5.0) were recommended to ^90^Y-DOTATOC therapy [51, 52]. For those NETs that demonstrate uptake in scintigraphy with ^111^In-octreotide, the therapy with ^111^In/^90^Y-octreotide is a modality [46].

^90^Y-DOTATOC is a potential choice which can deliver high absorbed doses to tumors expressing SST_2_ receptors, and the therapeutic response is achieved in about 25% of patients [23]. High-dose ^90^Y-DOTATOC targeted radiotherapy is a well-tolerated treatment which has significant clinical benefit and objective response for NETs [53]. A phase 2 study included 38 patients with advanced stage well-differentiated NETs treated with a fixed ^90^Y-DOTATOC dose of 2.56 GBq bimonthly showed that 43.6% patients had a partial response (PR), 25.6% had stable disease (SD), and 28.2% had progressive disease (PD) and that the median progression-free survival (PFS) was 22.3 months. The treatment of metastatic NETs with fixed activity is reported useful and safe [54].

Antitumor effects of  ^90^Y-DOTATOC have been reported considerably different between various studies [55]. A review revealed that the objective response rates are in the range from 20% to 28% for all NETs with ^90^Y-DOTATOC therapy. In patients with GEP-NET, the response rate was in the range from 28% to 38%. Overall, the cumulative response rate was 24% [56]. The objective responses were 5 PR, 7 minor responses, 29 SD, and 17 PD in a phase I dose-escalating treated study of ^90^Y-DOTATOC in 58 patients with SSTR-positive GEP-NET. Furthermore, there is a significantly longer overall survival (OS) compared with historic controls [57]. In metastatic NET patients, the result was complete response (CR) 4%, PR 23%, SD 62% in 116 patients, and PD 11%, and ^90^Y-DOTATOC also induced a better outcome [58]. Sowa-Staszczak et al. reported that ^90^Y-DOTATATE therapy results in symptomatic relief and tumor mass reduction in NETs. The response was 47% SD, 31% PR, and 9% PD, and the PFS was 37.4 months [59]. Clinical PR at six months was in 43 of 60 (72%) patients with histologically proven GEP-NETs after ^90^Y-DOTATATE treatment, and 9 patients had SD, and PD was noted in 8 patients. PFS was 17 months, and the OS was 22 months [49].

**Figure 2:**
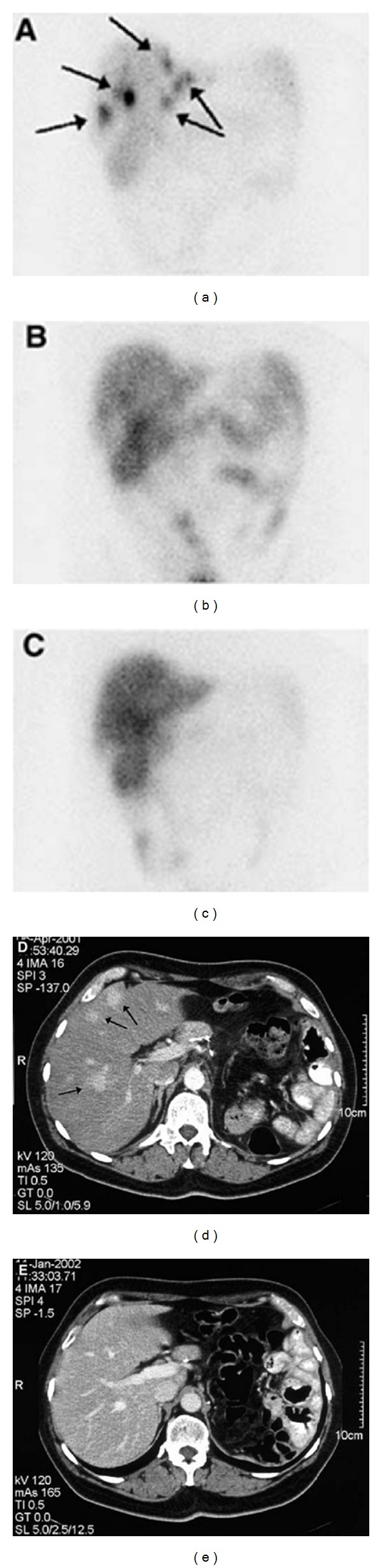
(a)–(c) Planar scans of the abdomen, 3 days after the injection of 200 mCi ^177^Lu-octreotate in a patient with liver metastases of an operated neuroendocrine pancreatic tumor. (a) After the first treatment; (b) after the second treatment; (c) after the fourth treatment. Note the loss of intensity of uptake in the liver lesions (arrows in (a)). This sign virtually always indicates a tumor volume response. (d) and (e). CT scans of the same patient: (d) before treatment; (e) 3 months after the last treatment. Tumor (arrows in (d)) is not demonstrated on (e). Neither MRI nor octreoscan could demonstrate definite tumor deposits at that time [66].

^177^Lu-DOTATATE, another radiopharmaceutical for treatment purpose, was used in GEP-NET patients. It was reported that CR and PR occurred in 2% and 28% of patients, respectively, and minor tumor response occurred in 16%. There was a 40 to 72 months survival benefit from diagnosis when compared with historical controls [60]. Treatment results with ^177^Lu-octreotate are preferable in patients with a limited lesion. Even in patients with no PD, early treatment may be better [47]. In the same patients, same dosage (3,700 MBq) of ^177^Lu-DOTATOC and ^177^Lu-DOTATATE was administered in different stages of treatment to see which should be preferred for PRRT. It indicated that the ^177^Lu-DOTATATE residence time of tumor was longer than ^177^Lu-DOTATOC [61]. This therapy is available, safe, and effective and has no serious adverse events [62]. ^177^Lu-DOTATATE is efficacious on small lesions when compared with ^90^Y-DOTATOC, which seems to be more efficient in bigger lesions [23, 63]; however, fractionated therapy with ^177^Lu-DOTATATE should be considered as a treatment option also for those patients with large tumors, high proliferation, and high receptor expression [64]. Studies with ^177^Lu-DOTATATE indicate that more cycles of such therapy are still safe. The median PFS is longer than 40 months [65]. The quality of life of those patients was improved remarkably after the therapy. Kwekkeboom et al. advocated ^177^Lu-octreotate therapy in patients with GEP tumors not waiting for tumor progression because of the high success rate and the absence of serious side effects (Figure 2) [66].

With ^177^Lu-DOTATATE treatments, tumor regression of 50% or more was achieved in 28% of patients. In 19% of patients, tumor regression was in 25% to 50%, SD showed in 35%, and PD showed in 18% of patients [55]. Quality of life is improved remarkably after treatment with ^177^Lu-DOTATATE [55, 67]. The combination of ^177^Lu-octreotate and capecitabine treatment was safe and feasible and may enhance these antitumor effects [68]. The study including 50 patients with metastasized NETs which compared combined ^90^Y/^177^Lu-DOTATATE therapy with single ^90^Y-DOTATATE showed that tandem radioisotopes therapy gives longer OS than a single one [69].

Oh et al. evaluated the effect of PRRT on the glucose metabolism and SSTR density assessed by ^18^F-FDG PET/CT and ^68^Ga-DOTANOC PET/CT, respectively. Only 56% (77/138) of the lesions show matched SSTR expression and glucose metabolism; the relationship is complicated [48]. In another study, the number of tumor lesions identified on ^177^Lu-DOTATATE scans during PRRT for dosage purpose was compared to those detected on ^68^Ga-DOTATATE studies obtained before the therapy; 318 lesions were detected in a total of 44 patients, while 280 (88%) lesions were concordant. Among those discordant lesions, 29 were ^68^Ga-DOTATATE positive and ^177^Lu-DOTATATE negative, whereas 9 were ^68^Ga-DOTATATE negative and ^177^Lu-DOTATATE positive. The sensitivity, accuracy, and positive predictive value for ^177^Lu-DOTA-TATE were 91%, 88%, and 97%, respectively, as compared to ^68^Ga-DOTATATE [70].

Radiolabeled octreotide analogues therapy is effective in patients with NETs [71], especially for GEP tumors [63]. The repeated cycles of PRRT enabled stabilization of the disease and did not cause an obvious toxicity increase of PRRT. Radiolabeled receptor-binding SST analogues (octreotide and lanreotide) target radioactivity to tissues expressing SSTRs which can be used for the management of NETs [63]. Side effects are described, and information on SST analog treatment is provided [72]. It is suggested that the octreotate PRRT is better when compared to octreotide in reducing diarrhea and flushing [61]. We summarize the studies that evaluate the PRRT efficacy in NETs in Table 3.

Dose-limiting factors for PRRT are kidney and/or bone marrow dose [61]. The uptake of ^68^Ga-DOTATOC was low in almost all organs except the kidneys [73]. The amount of radioactivity that can be used safely depends on the radiation dose to the kidneys [71]. The range of particles from ^90^Y is maximally 12 mm, which is long enough to reach the glomeruli; however, the range of the ^177^Lu electrons is shorter, maximally 2.1 mm, which causes much lower average decline in creatinine clearance in the latter patients than in the former patients [71]. The dose-limiting toxicity of ^90^Y-DOTATOC is renal insufficiency, starting at dose of 7.4 GBq/m^2^ [74]. However, the kidney and blood morphology parameters changes were transient [75]. When kidney protective agents are used, the side effects are few and mild [76]. PRRT therapy might become the first-line therapy in patients with disseminated or inoperable GEP-NETs [55]. The predictive factors for tumor remission include high tumor uptake on SRI and limited amount of liver metastases.

## 4. Somatostatin Receptor Based Reporter Gene Imaging

The human SSTR subtype 2 (hSSTr2), as a reporter gene, is under research for molecular imaging applications which have several features for potential translation to human studies [73, 77]. In vitro and in vivo studies have been done for this reporter system [73]. There are two approved SST analogues used for the expression of the reporter gene imaging [77]. SSTR2 is used as a reporter probe for imaging of gene transfer in animal models [78]. A study showed that the hSSTr2 cell membrane expression was proportional to the in vivo uptake of this radioligand demonstrated in tumor-bearing mice by small-animal PET of ^68^Ga-DOTATOC [73]. It is also verified by ^111^In-pentetreotide imaging that the ex vivo SST_2_ gene expression in tumor samples was positively related to the in vivo semiquantitative determination of SST_2_ protein [79]. ^94m^Tc-Demotate 1, an SST analog, was internalized rapidly into AdHASSTR2-infected A-427 cells, which will improve the sensitivity of the SSTR2 reporter gene system [78]. Briganti et al. studied nine neuroblastoma tumors with ^111^In-pentetreotide SPECT for SST_2_ and found that the ratio between the radioactivity in pathological and background area was increasing between early and late acquisitions. Moreover, the rate of this pathological increase was significantly related to the expression of SST_2_ gene [80].

The imaging of gene expression is critical to monitor gene transfer. There are great benefits for gene therapy trials from the use of noninvasive imaging to determine the location and time course of gene transfer [78]. Reporter transgenes with low endogenous expression levels are useful for this purpose [81]. ^111^In-octreotide detected the SSTR2 portion of the fusion protein in vivo (biodistribution studies and gamma-camera imaging) and in vitro (receptor-binding assay). This method can be used to monitor the delivery of a gene of interest directly and noninvasively [82]. Cotugno et al. used adeno-associated viral (AAV) vector-mediated gene transfer to murine muscle and liver which has low hSSTR2 expression and ^68^Ga-DOTATATE PET. They found that the levels of tracer accumulation correlated with the dosages of AAV vector used [81].

A study used a tumor model with an adenoviral vector encoding the human type 2 SSTR (Ad5-CMVhSSTr2) and a radiolabeled somatostatin-avid peptide (P829) to evaluate the level and location of the expression of the transferred gene [83]. Gene transfer technology can improve the degree and specificity of radiolabeled peptide localization in tumors [84]. The hSSTr2 was monitored as a reporter gene for ^99m^Tc-P2045 (an SST analogue) imaging showed adenoviral gene transfer to cancers, such as ovarian cancer [85]. This is a noninvasive imaging method for imaging gene transfer to ovarian cancer, which is helpful for planning a human gene therapy trial.

## 5. Discussion

Significant advances have been made in the imaging of NETs, but the challenge is to find the ideal imaging method with increased sensitivity and better tomographic localization of the primary and metastatic disease [86]. SRS is an ideal modality for evaluating NETs patients, which is not affected by their proliferative activity. Furthermore, when those patients have negative results on SRS, FDG PET should be used [87]. Both SPECT and PET can be very helpful in diagnosing NETs; however, PET may give more accurate information about the primary and metastatic lesions of NETs. PET or PET/CT is recommended as a first-line diagnostic imaging technology in patients with suspicious NETs [43]. The in vitro affinity of ^68^Ga-DOTATATE binding with the SST_2_ is higher than that of ^68^Ga-DOTATOC. However, the uptake value of the latter is higher than the former. The ^68^Ga-DOTATATE uptake and the histologic grade of NETs were not correlated [28]. Functional imaging with both ^68^Ga-DOTATATE and ^18^F-FDG is of potential use for a more comprehensive tumor assessment in intermediate-grade and high-grade tumors [33]. ^68^Ga-DOTATOC uptake in the head of the pancreas is commonly found in patients undergoing ^68^Ga-DOTATOC PET/CT. Therefore, quantification should be used to avoid false-positive diagnosis [88]. Furthermore, neither ^111^In-DTPAOC SPECT nor ^68^Ga-DOTATOC PET imaging was sensitive in the detection of liver metastases since they showed a lower uptake than the surrounding normal liver tissue compared to CT [35]. Recently, a new ^11^C-5-HTP-PET has been reported that is sensitive in small NET lesions imaging and can image more tumor lesions than SRI and CT [89]. Moreover, scintigraphy of the upper abdomen is affected by breathing artifacts, so misalignment due to respiratory motions must be considered [88].

Despite the fact that most GEP-NETs are slow growing, OS in NET patients with liver metastases is 2 to 4 years. In metastatic cases, there are limits of cytoreductive therapeutic options [60]. PRRT is a promising new method in the treatment of patients with inoperable or metastasized NETs because of its fewer side effects and less toxicity and better curative effect [86]. Individual dosimetry seems helpful for deciding whether a patient can be chosen for radiolabeled DOTATOC or DOTATATE therapy or not and deciding the therapeutic modality for each patient [32]. Evaluation of NETs therapy response is difficult; for assessing such response, the monitoring of functional parameters is more accurate than morphologic measurements [45]. A positive scintigram suggests good response to treatment with octreotide in many cases [19]. The foundation of the dose-response relationship and the decision of the correct dose of PRRT are important to achieve an ideal treatment [56].

Beta particles with higher energies and longer range emitted by ^90^Y may be preferable for larger tumors, while ^177^Lu that emits beta particles with shorter range and longer half-life may be a good choice for small tumors. In patients with tumors of nonhomogeneous receptor distribution and various sizes, a combination of radionuclide might be useful [69]. ^90^Y-DOTATOC therapy has proven to be an effective and safe treatment. Before and after the therapy, blood tests for kidney, liver function, and chromogranin A were performed. During 12 months followup, transient decrease of PLT, WBC, and hemoglobin values and GFR values were found [59]. The mild critical organ toxicity does not limit the PRRT of NETs [59]. Standard dosages of ^90^Y-DOTATATE result in a relatively low risk of myelotoxicity. However, because of risk of renal toxicity, the kidneys shoud be monitored carefully [49]. The further goal is to further reduce renal toxicity so that higher doses can be administered [53].

^68^Ga-DOTATOC is a specific ligand for hSSTr2 reporter system and so that for hSSTr2 reporter gene PET imaging. Because DOTATOC has been tested clinically, this reporter system can be used for translation to human studies [73]. The relative level of gene expression for SST_2_ was positively related to patient outcome in the childhood neuroblastoma tumor and neuroblastoma tumor. Imaging with ^111^In-pentetreotide may have not only a diagnostic but also a prognostic value [80]. There is great use of ^99m^Tc-labeled peptides for imaging gene transfer with the hSSTr2 reporter receptor, specifically when the reporter correlates with the expression of therapeutic genes [83]. Selective internal radiation therapy (SIRT) is a well tolerated and effective treatment for nonresectable NET with liver metastases [90].

## 6. Perspective

The SSTR-based molecular imaging is a noninvasive and quantitative method to diagnose the NETs and evaluate the therapeutic efficacy for NETs. The development of radiolabeled SST analogues has affected the clinical management of patients with NETs. PET/CT can be useful in the early prediction of the treatment outcome of NET patients who underwent PRRT. Furthermore, the clinical management of NETs will be further improved if better radioligands are developed and more technologies are used to identify the radiotherapy treatment response in patients with NETs.

Dual therapy is a promising method to treat NETs. The combination of PRRT and EBRT can increase the dose delivered to the tumor and reduce the dose for organs at risk. The clinical use of molecular imaging is not only in diagnosis and treatment efficacy evaluation, but also in patient selection. Still, it plays an important role in the reporter gene research. Personalized diagnosis and treatment of NETs will be established based on increased understanding of molecular mechanisms of NETs.

## Figures and Tables

**Table 1:** Characteristics of radionuclides used for SRI and PRRT.

Radionuclide	Type of decay	Type of rays	Half-life	Energy	Producer
^ 111^In	EC	*γ*	2.8 days	173 KeV	^ 111^Cd (p, n)
				247 KeV	
^ 18^F	*β*+	*β*	109.8 min	511 KeV	^ 20^Ne (d, *α*)
	EC				^ 18^O (p, n)
^ 68^Ga	*β*+	*β*	68.3 min	511 KeV	^ 68^Ge-^68^Ga generator
^ 90^Y	*β*−	*β*	64 h	2.288 MeV	^ 90^Sr-^90^Y generator
^ 177^Lu	*β*−	*β*	6.7 days	0.5 MeV	^ 176^Lu (n, *γ*)
					^ 176^Yb (n, *γ*)
^ 64^Cu	*β*−	*β*	12.7 h	0.58 MeV	^ 63^Cu (n, *γ*)
	*β*+			0.653 MeV	^ 64^Zn (n, p)
	EC	*γ*		1.346 MeV	

**Table 2:** The tracer used for SPECT and PET in NETs and for gene imaging.

Types of imaging	Radiotracer
SPECT	^ 111^In-pentetreotide
	^ 111^In-DTPAOC
	^ 123^I-octreotide
	^ 111^In-DOTA-lanreotide
	^ 111^In-DOTA-NOC-ATE
	^ 111^In-DOTA-BOC-ATE

PET	^ 68^Ga-DOTATATE
	^ 68^Ga-DOTATOC
	^ 68^Ga-DOTANOC
	^ 64^Cu-DOTATATE
	^ 18^F-FP-Gluc-TOCA

Gene imaging	^ 94m^Tc-Demotate 1
	^ 99m^Tc-P2045
	^ 99m^Tc-P829

**Table 3:** The radioagent used in PRRT and the efficacy of the therapy.

Therapeutic agents	Subjects	Dosage	Duration	Main findings	References
^ 90^Y-DOTATATE	46 NETs	7.4 GBq/m^2^	3–5 cycles	PFS 37.4 months	[59]
^ 90^Y-DOTATOC	116 Metastatic NETs	162–200 mCi/m^2^	2–4 cycles	Significant reduction of symptoms was found in 83% of patients	[58]
^ 177^Lu-DOTATATE	310GEP-NETs	750 to 800 mCi	4 cycles	Survival benefit of 40 to 72 months from diagnosis	[60]
^ 177^Lu-DOTATOC	27 relapse NETs	7,400 MBq	Once	2 PR, 5 MR, 12 SD, and 8 PD	[62]

## Abbreviations

NETs: Neuroendocrine tumors
GEP: Gastroenteropancrea
SSTR: Somatostatin receptors
SST: Somatostatin
SST_2_: Sst subtype 2
SRI: Somatostatin receptor imaging
SPECT: Single photon emission computed tomography
^111^In-DTPAOC: [^111^In-DTPA(0)]octreotide
PET: Positron emission tomography
^68^Ga-DOTATATE: ^68^Ga-DOTA(0), Tyr(3)octreotate
SRS: Somatostatin receptor scintigraphy
CT: Computed tomography
PRRT: Peptide receptor radionuclide therapy
SUV: Standard uptake value
^18^F-FP-Gluc-TOCA: ^18^F-fluoropropionyl-Lys0-Tyr3-octreotate
SRTRT: Somatostatin receptor targeted radionuclide therapy
RECIST: Response evaluation criteria in solid tumors
PR: Partial response
SD: Stable disease
PD: Progressive disease
PFS: Progression-free survival
OS: Overall survival
CR: Complete response
hSSTr2: Human somatostatin receptor subtype 2
AAV: Adeno-associated viral
SIRT: Selective internal radiation therapy.

## References

[1] Koopmans KP, Neels ON, Kema IP (2009). Molecular imaging in neuroendocrine tumors: molecular uptake mechanisms and clinical results. *Critical Reviews in Oncology/Hematology*.

[2] Rufini V, Calcagni ML, Baum RP (2006). Imaging of neuroendocrine tumors. *Seminars in Nuclear Medicine*.

[3] Capella C, Heirz PU, Hofler H, Solcia E, Kloppel G (1995). Revised classification of neuroendocrine tumours of the lung, pancreas and gut. *Virchows Archiv*.

[4] Bombardieri E, Maccauro M, De Deckere E, Savelli G, Chiti A (2001). Nuclear medicine imaging of neuroendocrine tumours. *Annals of Oncology*.

[5] Ambrosini V, Campana D, Tomassetti P, Grassetto G, Rubello D, Fanti S (2011). PET/CT with ^68^Gallium-DOTA-peptides in NET: an overview. *European Journal of Radiology*.

[6] Stoyianni A, Pentheroudakis G, Pavlidis N (2011). Neuroendocrine carcinoma of unknown primary: a systematic review of the literature and a comparative study with other neuroendocrine tumors. *Cancer Treatment Reviews*.

[7] Modlin IM, Lye KD, Kidd M (2003). A 5-decade analysis of 13,715 carcinoid tumors. *Cancer*.

[8] Lindholm DP, Oberg K (2011). Biomarkers and molecular imaging in gastroenteropancreatic neuroendocrine tumors. *Hormone and Metabolic Research*.

[9] Oberg K (1994). Biology, diagnosis, and treatment of neuroendocrine tumors of the gastrointestinal tract. *Current Opinion in Oncology*.

[10] Strong VE, Kennedy T, Al-Ahmadie H (2008). Prognostic indicators of malignancy in adrenal pheochromocytomas: clinical, histopathologic, and cell cycle/apoptosis gene expression analysis. *Surgery*.

[11] Pape U-F, Berndt U, Müller-Nordhorn J (2008). Prognostic factors of long-term outcome in gastroenteropancreatic neuroendocrine tumours. *Endocrine-Related Cancer*.

[12] Sundin A, Rockall A (2012). Therapeutic monitoring of gastroenteropancreatic neuroendocrine tumors: the challenges ahead. *Neuroendocrinology*.

[13] Patel YC (1999). Somatostatin and its receptor family. *Frontiers in Neuroendocrinology*.

[14] Brazeau P, Epelbaum J, Tannenbaum GS (1978). Somatostatin: isolation, characterization, distribution, and blood determination. *Metabolism*.

[15] Hoyer D, Bell GI, Berelowitz M (1995). Classification and nomenclature of somatostatin receptors. *Trends in Pharmacological Sciences*.

[16] Reubi JC, Waser B, Schaer J-C, Laissue JA (2001). Somatostatin receptor sst1-sst5 expression in normal and neoplastic human tissues using receptor autoradiography with subtype-selective ligands. *European Journal of Nuclear Medicine*.

[17] Lamberts SWJ, Bakker WH, Reubi J-C, Krenning EP (1990). Somatostatin-receptor imaging in the localization of endocrine tumors. *The New England Journal of Medicine*.

[18] Reubi JC, Kvols L, Krenning E, Lamberts SWJ (1990). Distribution of somatostatin receptors in normal and tumor tissue. *Metabolism*.

[19] Krenning EP, Kwekkeboom DJ, Bakker WH (1993). Somatostatin receptor scintigraphy with [^111^In-DTPA-D-Phe^1^]- and [^123^I-Tyr^3^]-octreotide: the Rotterdam experience with more than 1000 patients. *European Journal of Nuclear Medicine*.

[20] Reubi JC, Maecke HR (2008). Peptide-based probes for cancer imaging. *Journal of Nuclear Medicine*.

[21] De Herder WW, Hofland LJ, Van Der Lely AJ, Lamberts SWJ (2003). Somatostatin receptors in gastroentero-pancreatic neuroendocrine tumours. *Endocrine-Related Cancer*.

[22] Papotti M, Bongiovanni M, Volante M (2002). Expression of somatostatin receptor types 1–5 in 81 cases of gastrointestinal and pancreatic endocrine tumors: a correlative immunohistochemical and reverse-transcriptase polymerase chain reaction analysis. *Virchows Archiv*.

[23] Bodei L, Cremonesi M, Grana C (2004). Receptor radionuclide therapy with ^90^Y-[DOTA]^0^-Tyr^3^-octreotide (^90^Y-DOTATOC) in neuroendocrine tumours. *European Journal of Nuclear Medicine and Molecular Imaging*.

[24] Al-Nahhas A, Win Z, Szyszko T (2007). Gallium-68 PET: a new frontier in receptor cancer imaging. *Anticancer Research*.

[25] Wong KK, Waterfield RT, Marzola MC (2012). Contemporary nuclear medicine imaging of neuroendocrine tumours. *Clinical Radiology*.

[26] Naswa N, Bal CS (2012). Divergent role of ^68^Ga-labeled somatostatin analogs in the workup of patients with NETs: AIIMS experience. *Recent Results in Cancer Research*.

[27] de Herder WW, Kwekkeboom DJ, Feelders RA (2006). Somatostatin receptor imaging for neuroendocrine tumors. *Pituitary*.

[28] Srirajaskanthan R, Kayani I, Quigley AM, Soh J, Caplin ME, Bomanji J (2010). The role of ^68^Ga-DOTATATE PET in patients with neuroendocrine tumors and negative or equivocal findings on ^111^In-DTPA-octreotide scintigraphy. *Journal of Nuclear Medicine*.

[29] De Jong M, Bakker WH, Krenning EP (1997). Yttrium-90 and indium-111 labelling, receptor binding and biodistribution of [DOTA^0^,D-Phe^1^,Tyr^3^]octreotide, a promising somatostatin analogue for radionuclide therapy. *European Journal of Nuclear Medicine*.

[30] Kwekkeboom DJ, Krenning EP (2002). Somatostatin receptor imaging. *Seminars in Nuclear Medicine*.

[31] Schmidt M, Fischer E, Dietlein M (2002). Clinical value of somatostatin receptor imaging in patients with suspected head and neck paragangliomas. *European Journal of Nuclear Medicine*.

[32] Poeppel TD, Binse I, Petersenn S (2011). ^68^Ga-DOTATOC versus ^68^Ga-DOTATATE PET/CT in functional imaging of neuroendocrine tumors. *Journal of Nuclear Medicine*.

[33] Kayani I, Bomanji JB, Groves A (2008). Functional imaging of neuroendocrine tumors with combined PET/CT using ^68^Ga-DOTATATE (Dota-DPhe^1^, Tyr^3^-octreotate) and ^18^F-FDG. *Cancer*.

[34] Kayani I, Conry BG, Groves AM (2009). A comparison of ^68^Ga-DOTATATE and ^18^F-FDG PET/CT in pulmonary neuroendocrine tumors. *Journal of Nuclear Medicine*.

[35] Kowalski J, Henze M, Schuhmacher J, Mäcke HR, Hofmann M, Haberkorn U (2003). Evaluation of positron emission tomography imaging using [^68^Ga]-DOTA-D Phe^1^-Tyr^3^-octreotidein comparison to [^111^In]-DTPAOC SPECT. First results in patients with neuroendocrine tumors. *Molecular Imaging and Biology*.

[36] Gabriel M, Decristoforo C, Kendler D (2007). ^68^Ga-DOTA-Tyr^3^-octreotide PET in neuroendocrine tumors: comparison with somatostatin receptor scintigraphy and CT. *Journal of Nuclear Medicine*.

[37] Poeppel TD, Binse I, Petersenn S (2011). ^68^Ga-DOTATOC versus ^68^Ga-DOTATATE PET/CT in functional imaging of neuroendocrine tumors. *Journal of Nuclear Medicine*.

[38] Desai K, Watkins J, Woodward N (2011). Use of molecular imaging to differentiate liver metastasis of colorectal cancer metastasis from neuroendocrine tumor origin. *Journal of Clinical Gastroenterology*.

[39] Wild D, Mäcke HR, Waser B (2005). ^68^Ga-DOTANOC: a first compound for PET imaging with high affinity for somatostatin receptor subtypes 2 and 5. *European Journal of Nuclear Medicine and Molecular Imaging*.

[40] Pfeifer A, Knigge U, Mortensen J (2012). Clinical PET of neuroendocrine tumors using ^64^Cu-DOTATATE: first-in-humans study. *Journal of Nuclear Medicine*.

[41] Wester HJ, Schottelius M, Scheidhauer K (2003). PET imaging of somatostatin receptors: design, synthesis and preclinical evaluation of a novel ^18^F-labelled, carbohydrated analogue of octreotide. *European Journal of Nuclear Medicine and Molecular Imaging*.

[42] Meisetschläger G, Poethko T, Stah A (2006). Gluc-Lys([^18^F]FP)-TOCA PET in patients with SSTR-positive tumors: biodistribution and diagnostic evaluation compared with [^111^In]DTPA-octreotide. *Journal of Nuclear Medicine*.

[43] Treglia G, Castaldi P, Rindi G, Giordano A, Rufini V (2012). Diagnostic performance of Gallium-68 somatostatin receptor PET and PET/CT in patients with thoracic and gastroenteropancreatic neuroendocrine tumours: a meta-analysis. *Endocrine*.

[44] Ambrosini V, Campana D, Bodei L (2010). ^68^Ga-DOTANOC PET/CT clinical impact in patients with neuroendocrine tumors. *Journal of Nuclear Medicine*.

[45] Haug AR, Auernhammer CJ, Wängler B (2010). ^68^Ga-DOTATATE PET/CT for the early prediction of response to somatostatin receptor-mediated radionuclide therapy in patients with well-differentiated neuroendocrine tumors. *Journal of Nuclear Medicine*.

[46] Kaltsas G, Rockall A, Papadogias D, Reznek R, Grossman AB (2004). Recent advances in radiological and radionuclide imaging and therapy of neuroendocrine tumours. *European Journal of Endocrinology*.

[47] Kwekkeboom DJ, Teunissen JJ, Bakker WH (2005). Radiolabeled somatostatin analog [^177^Lu-DOTA^0^, Tyr^3^]octreotate in patients with endocrine gastroenteropancreatic tumors. *Journal of Clinical Oncology*.

[48] Oh S, Prasad V, Lee DS, Baum RP (2011). Effect of peptide receptor radionuclide therapy on somatostatin receptor status and glucose metabolism in neuroendocrine tumors: intraindividual comparison of Ga-68 DOTANOC PET/CT and F-18FDG PET/CT. *International Journal of Molecular Imaging*.

[49] Cwikla JB, Sankowski A, Seklecka N (2009). Efficacy of radionuclide treatment DOTATATE Y-90 in patients with progressive metastatic gastroenteropancreatic neuroendocrine carcinomas (GEP-NETs): a phase II study. *Annals of Oncology*.

[50] Ezziddin S, Lohmar J, Yong-Hing CJ (2012). Does the pretherapeutic tumor SUV in ^68^Ga DOTATOC PET predict the absorbed dose of ^177^Lu octreotate?. *Clinical Nuclear Medicine*.

[51] Koukouraki S, Strauss LG, Georgoulias V, Eisenhut M, Haberkorn U, Dimitrakopoulou-Strauss A (2006). Comparison of the pharmacokinetics of ^68^Ga-DOTATOC and [^18^F]FDG in patients with metastatic neuroendocrine tumours scheduled for ^90^Y-DOTATOC therapy. *European Journal of Nuclear Medicine and Molecular Imaging*.

[52] Koukouraki S, Strauss LG, Georgoulias V (2006). Evaluation of the pharmacokinetics of ^68^Ga-DOTATOC in patients with metastatic neuroendocrine tumours scheduled for ^90^Y-DOTATOC therapy. *European Journal of Nuclear Medicine and Molecular Imaging*.

[53] Waldherr C, Pless M, Maecke HR (2002). Tumor response and clinical benefit in neuroendocrine tumors after 7.4 Gbq ^90^Y-DOTATOC. *Journal of Nuclear Medicine*.

[54] Savelli G, Bertagna F, Franco F (2012). Final results of a phase 2A study for the treatment of metastatic neuroendocrine tumors with a fixed activity of ^90^Y-DOTA-D-Phe^1^-Tyr^3^ octreotide. *Cancer*.

[55] Van Essen M, Krenning EP, De Jong M, Valkema R, Kwekkeboom DJ (2007). Peptide Receptor Radionuclide Therapy with radiolabelled somatostatin analogues in patients with somatostatin receptor positive tumours. *Acta Oncologica*.

[56] Nisa L, Savelli G, Giubbini R (2011). Yttrium-90 DOTATOC therapy in GEP-NET and other SST2 expressing tumors: a selected review. *Annals of Nuclear Medicine*.

[57] Valkema R, Pauwels S, Kvols LK (2006). Survival and response after peptide receptor radionuclide therapy with [^90^Y-DOTA^0^,Tyr^3^]octreotide in patients with advanced gastroenteropancreatic neuroendocrine tumors. *Seminars in Nuclear Medicine*.

[58] Forrer F, Waldherr C, Maecke HR, Mueller-Brand J (2006). Targeted radionuclide therapy with ^90^Y-DOTATOC in patients with neuroendocrine tumors. *Anticancer Research*.

[59] Sowa-Staszczak A, Pach D, Kunikowska J (2011). Efficacy and safety of ^90^Y-DOTATATE therapy in neuroendocrine tumours. *Endokrynologia Polska*.

[60] Kwekkeboom DJ, De Herder WW, Kam BL (2008). Treatment with the radiolabeled somatostatin analog [^177^Lu- DOTA^0^,Tyr^3^]octreotate: toxicity, efficacy, and survival. *Journal of Clinical Oncology*.

[61] Esser JP, Krenning EP, Teunissen JJM (2006). Comparison of [^177^Lu-DOTA^0^,Tyr^3^]octreotate and [^177^Lu-DOTA^0^,Tyr^3^]octreotide: which peptide is preferable for PRRT?. *European Journal of Nuclear Medicine and Molecular Imaging*.

[62] Forrer F, Uusijärvi H, Storch D, Maecke HR, Mueller-Brand J (2005). Treatment with ^177^Lu-DOTATOC of patients with relapse of neuroendocrine tumors after treatment with ^90^Y-DOTATOC. *Journal of Nuclear Medicine*.

[63] Kaltsas GA, Papadogias D, Makras P, Grossman AB (2005). Treatment of advanced neuroendocrine tumours with radiolabelled somatostatin analogues. *Endocrine-Related Cancer*.

[64] Garske U, Sandstrom M, Johansson S (2012). Lessons on tumour response: imaging during therapy with ^177^Lu-DOTA-octreotate. a case report on a patient with a large volume of poorly differentiated neuroendocrine carcinoma. *Theranostics*.

[65] Kam BL, Teunissen JJ, Krenning EP (2012). Lutetium-labelled peptides for therapy of neuroendocrine tumours. *European Journal of Nuclear Medicine and Molecular Imaging*.

[66] Kwekkeboom DJ, Bakker WH, Kam BL (2003). Treatment of patients with gastro-entero-pancreatic (GEP) tumours with the novel radiolabelled somatostatin analogue [^177^Lu-DOTA^0^,Tyr^3^]octreotate. *European Journal of Nuclear Medicine and Molecular Imaging*.

[67] Kwekkeboom DJ, de Herder WW, van Eijck CHJ (2010). Peptide receptor radionuclide therapy in patients with gastroenteropancreatic neuroendocrine tumors. *Seminars in Nuclear Medicine*.

[68] Van Essen M, Krenning EP, Kam BL, De Herder WW, Van Aken MO, Kwekkeboom DJ (2008). Report on short-term side effects of treatments with ^177^Lu- octreotate in combination with capecitabine in seven patients with gastroenteropancreatic neuroendocrine tumours. *European Journal of Nuclear Medicine and Molecular Imaging*.

[69] Kunikowska J, Królicki L, Hubalewska-Dydejczyk A, Mikolajczak R, Sowa-Staszczak A, Pawlak D (2011). Clinical results of radionuclide therapy of neuroendocrine tumours with ^90^Y-DOTATATE and tandem^90^ Y/^177^Lu-DOTATATE: which is a better therapy option?. *European Journal of Nuclear Medicine and Molecular Imaging*.

[70] Sainz-Esteban A, Prasad V, Schuchardt C, Zachert C, Carril JM, Baum RP (2012). Comparison of sequential planar ^177^Lu-DOTA-TATE dosimetry scans with ^68^Ga-DOTA-TATE PET/CT images in patients with metastasized neuroendocrine tumours undergoing peptide receptor radionuclide therapy. *European Journal of Nuclear Medicine and Molecular Imaging*.

[71] Valkema R, Pauwels SA, Kvols LK (2005). Long-term follow-up of renal function after peptide receptor radiation therapy with ^90^Y-DOTA^0^,Tyr^3^-octreotide and ^177^Lu-DOTA^0^,Tyr^3^-octreotate. *Journal of Nuclear Medicine*.

[72] Öberg K, Kvols L, Caplin M (2004). Consensus report on the use of somatostatin analogs for the management of neuroendocrine tumors of the gastroenteropancreatic system. *Annals of Oncology*.

[73] Zhang H, Moroz MA, Serganova I (2011). Imaging expression of the human somatostatin receptor subtype-2 reporter gene with ^68^Ga-DOTATOC. *Journal of Nuclear Medicine*.

[74] Cremonesi M, Ferrari M, Zoboli S (1999). Biokinetics and dosimetry in patients administered with ^111^In-DOTA-Tyr^3^-octreotide: implications for internal radiotherapy with ^90^Y-DOTATOC. *European Journal of Nuclear Medicine*.

[75] Pach D, Sowa-Staszczak A, Kunikowska J (2012). Repeated cycles of peptide receptor radionuclide therapy (PRRT)—results and side-effects of the radioisotope ^90^Y-DOTA TATE,^177^Lu-DOTA TATE or ^90^Y/^177^Lu-DOTA TATE therapy in patients with disseminated NET. *Radiotherapy and Oncology*.

[76] Kwekkeboom DJ, Kam BL, Van Essen M (2010). Somatostatin receptor-based imaging and therapy of gastroenteropancreatic neuroendocrine tumors. *Endocrine-Related Cancer*.

[77] Zinn KR, Chaudhuri TR (2002). The type 2 human somatostatin receptor as a platform for reporter gene imaging. *European Journal of Nuclear Medicine*.

[78] Rogers BE, Parry JJ, Andrews R, Cordopatis P, Nock BA, Maina T (2005). MicroPET imaging of gene transfer with a somatostatin receptor-based reporter gene and 94mTc-demotate 1. *Journal of Nuclear Medicine*.

[79] Orlando C, Raggi CC, Bagnoni L, Sestini R, Briganti V, La Cava G (2001). Somatostatin receptor type 2 gene expression in neuroblastoma, measured by competitive RT-PCR, is related to patient survival and to somatostatin receptor imaging by indium -111-pentetreotide. *Medical and Pediatric Oncology*.

[80] Briganti V, Sestini R, Orlando C (1997). Imaging of somatostatin receptors by indium-111-pentetreotide correlates with quantitative determination of somatostatin receptor type 2 gene expression in neuroblastoma tumors. *Clinical Cancer Research*.

[81] Cotugno G, Aurilio M, Annunziata P (2011). Noninvasive repetitive imaging of somatostatin receptor 2 gene transfer with positron emission tomography. *Human Gene Therapy*.

[82] Kundra V, Mannting F, Jones AG, Kassis AI (2002). Noninvasive monitoring of somatostatin receptor type 2 chimeric gene transfer. *Journal of Nuclear Medicine*.

[83] Zinn KR, Buchsbaum DJ, Chaudhuri TR, Mountz JM, Grizzle WE, Rogers BE (2000). Noninvasive monitoring of gene transfer using a reporter receptor imaged with a high-affinity peptide radiolabeled with 99mTc or 188Re. *Journal of Nuclear Medicine*.

[84] Buchsbaum DJ, Chaudhuri TR, Yamamoto M, Zinn KR (2004). Gene expression imaging with radiolabeled peptides. *Annals of Nuclear Medicine*.

[85] Chaudhuri TR, Rogers BE, Buchsbaum DJ, Mountz JM, Zinn KR (2001). A noninvasive reporter system to image adenoviral-mediated gene transfer to ovarian cancer xenografts. *Gynecologic Oncology*.

[86] Teunissen JJM, Kwekkeboom DJ, Valkema R, Krenning EP (2011). Nuclear medicine techniques for the imaging and treatment of neuroendocrine tumours. *Endocrine-Related Cancer*.

[87] Belhocine T, Foidart J, Rigo P (2002). Fluorodeoxyglucose positron emission tomography and somatostatin receptor scintigraphy for diagnosing and staging carcinoid tumours: correlations with the pathological indexes p53 and Ki-67. *Nuclear Medicine Communications*.

[88] Al-Ibraheem A, Bundschuh RA, Notni J (2011). Focal uptake of ^68^Ga-DOTATOC in the pancreas: pathological or physiological correlate in patients with neuroendocrine tumours?. *European Journal of Nuclear Medicine and Molecular Imaging*.

[89] Orlefors H, Sundin A, Garske U (2005). Whole-body 11C-5-hydroxytryptophan positron emission tomography as a universal imaging technique for neuroendocrine tumors: comparison with somatostatin receptor scintigraphy and computed tomography. *Journal of Clinical Endocrinology and Metabolism*.

[90] Rajekar H, Bogammana K, Stubbs RS (2011). Selective internal radiation therapy for gastrointestinal neuroendocrine tumour liver metastases: a new and effective modality for treatment. *International Journal of Hepatology*.

